# Assessing the landscape of *STXBP1*-related disorders in 534 individuals

**DOI:** 10.1093/brain/awab327

**Published:** 2021-11-23

**Authors:** Julie Xian, Shridhar Parthasarathy, Sarah M Ruggiero, Ganna Balagura, Eryn Fitch, Katherine Helbig, Jing Gan, Shiva Ganesan, Michael C Kaufman, Colin A Ellis, David Lewis-Smith, Peter Galer, Kristin Cunningham, Margaret O’Brien, Mahgenn Cosico, Kate Baker, Alejandra Darling, Fernanda Veiga de Goes, Christelle M El Achkar, Jan Henje Doering, Francesca Furia, Ángeles García-Cazorla, Elena Gardella, Lisa Geertjens, Courtney Klein, Anna Kolesnik-Taylor, Hanna Lammertse, Jeehun Lee, Alexandra Mackie, Mala Misra-Isrie, Heather Olson, Emma Sexton, Beth Sheidley, Lacey Smith, Luiza Sotero, Hannah Stamberger, Steffen Syrbe, Kim Marie Thalwitzer, Annemiek van Berkel, Mieke van Haelst, Christopher Yuskaitis, Sarah Weckhuysen, Ben Prosser, Charlene Son Rigby, Scott Demarest, Samuel Pierce, Yuehua Zhang, Rikke S Møller, Hilgo Bruining, Annapurna Poduri, Federico Zara, Matthijs Verhage, Pasquale Striano, Ingo Helbig

**Affiliations:** 1 Division of Neurology, Children’s Hospital of Philadelphia, Philadelphia, PA 19104, USA; 2 The Epilepsy NeuroGenetics Initiative (ENGIN), Children's Hospital of Philadelphia, Philadelphia, PA 19104, USA; 3 Department of Biomedical and Health Informatics (DBHi), Children’s Hospital of Philadelphia, Philadelphia, PA 19146, USA; 4 Neuroscience Program, University of Pennsylvania, Philadelphia, PA 19104, USA; 5 Department of Biology, The College of New Jersey, Ewing Township, NJ 08618, USA; 6 Department of Neurosciences, Rehabilitation, Ophthalmology, Genetics, and Maternal and Child Health, University of Genoa, Genoa, Italy; 7 Pediatric Neurology and Muscular Diseases Unit, IRCCS ‘G. Gaslini’ Institute, Genoa, Italy; 8 Department of Pediatrics, West China Second University Hospital, Sichuan University, Chengdu, China; 9 Key Laboratory of Birth Defects and Related Diseases of Women and Children (Sichuan University), Ministry of Education, Chengdu, China; 10 Department of Neurology, University of Pennsylvania Perelman School of Medicine, Philadelphia, PA 19104, USA; 11 Translational and Clinical Research Institute, Newcastle University, Newcastle-upon-Tyne NE2 4HH, UK; 12 Royal Victoria Infirmary, Newcastle-upon-Tyne NE1 4LP, UK; 13 Center for Neuroengineering and Therapeutics, University of Pennsylvania, Philadelphia, PA 19104, USA; 14 Lewis Katz School of Medicine, Temple University, Philadelphia, PA 19140, USA; 15 MRC Cognition and Brain Sciences Unit, University of Cambridge, Cambridge, UK; 16 Pediatric Neurology Department, Hospital Sant Joan de Déu, University of Barcelona, Barcelona, Spain; 17 Department of Pediatrics and Pediatric Neurology Laboratory, Instituto Fernandes Figueira, Rio de Janeiro 22250-020, Brazil; 18 Division of Epilepsy and Clinical Neurophysiology and Epilepsy Genetics Program, Department of Neurology, Boston Children's Hospital, Boston, MA, USA; 19 Division of Pediatric Epileptology, Centre for Pediatric and Adolescent Medicine, University Hospital Heidelberg, 69120 Heidelberg, Germany; 20 Department of Clinical Neurophysiology, Danish Epilepsy Center Filadelfia, Dianalund 4293, Denmark; 21 Department of Child and Adolescent Psychiatry, Amsterdam UMC, University of Amsterdam, Amsterdam, The Netherlands; 22 Departments of Pediatrics and Neurology, Children's Hospital Colorado, Aurora, CO 80045, USA; 23 Department of Human Genetics, Center for Neurogenomics and Cognitive Research (CNCR), Amsterdam University Medical Center, de Boelelaan 1085, 1081 HV Amsterdam, The Netherlands; 24 Department of Pediatrics, Samsung Medical Center, School of Medicine, Sungkyunkwan University, Seoul, Republic of Korea; 25 Division of Neurology, University Hospital Antwerp, Antwerp, Belgium; 26 Applied & Translational Neurogenomics Group, VIB Center for Molecular Neurology, VIB, Antwerp, Belgium; 27 Department of Functional Genomics, Center for Neurogenomics and Cognitive Research (CNCR), VU University Amsterdam, De Boelelaan 1085, 1081 HV Amsterdam, The Netherlands; 28 Translational Neurosciences, Faculty of Medicine and Health Science, University of Antwerp, Antwerp, Belgium; 29 Department of Physiology, University of Pennsylvania Perelman School of Medicine, Philadelphia, PA 19104, USA; 30 STXBP1 Foundation, Apex, NC 27539, USA; 31 Department of Pediatrics, Beijing University First Hospital, Beijing, China; 32 Unit of Medical Genetics, IRCCS Istituto Giannina Gaslini, Genova, Italy

**Keywords:** *STXBP1*, epilepsy, genetics, developmental and epileptic encephalopathy, Human Phenotype Ontology

## Abstract

Disease-causing variants in *STXBP1* are among the most common genetic causes of neurodevelopmental disorders. However, the phenotypic spectrum in *STXBP1*-related disorders is wide and clear correlations between variant type and clinical features have not been observed so far.

Here, we harmonized clinical data across 534 individuals with *STXBP1*-related disorders and analysed 19 973 derived phenotypic terms, including phenotypes of 253 individuals previously unreported in the scientific literature. The overall phenotypic landscape in *STXBP1*-related disorders is characterized by neurodevelopmental abnormalities in 95% and seizures in 89% of individuals, including focal-onset seizures as the most common seizure type (47%). More than 88% of individuals with *STXBP1*-related disorders have seizure onset in the first year of life, including neonatal seizure onset in 47%. Individuals with protein-truncating variants and deletions in *STXBP1* (*n* = 261) were almost twice as likely to present with West syndrome and were more phenotypically similar than expected by chance. Five genetic hotspots with recurrent variants were identified in more than 10 individuals, including p.Arg406Cys/His (*n* = 40), p.Arg292Cys/His/Leu/Pro (*n* = 30), p.Arg551Cys/Gly/His/Leu (*n* = 24), p.Pro139Leu (*n* = 12), and p.Arg190Trp (*n* = 11). None of the recurrent variants were significantly associated with distinct electroclinical syndromes, single phenotypic features, or showed overall clinical similarity, indicating that the baseline variability in *STXBP1*-related disorders is too high for discrete phenotypic subgroups to emerge. We then reconstructed the seizure history in 62 individuals with *STXBP1*-related disorders in detail, retrospectively assigning seizure type and seizure frequency monthly across 4433 time intervals, and retrieved 251 anti-seizure medication prescriptions from the electronic medical records. We demonstrate a dynamic pattern of seizure control and complex interplay with response to specific medications particularly in the first year of life when seizures in *STXBP1*-related disorders are the most prominent. Adrenocorticotropic hormone and phenobarbital were more likely to initially reduce seizure frequency in infantile spasms and focal seizures compared to other treatment options, while the ketogenic diet was most effective in maintaining seizure freedom.

In summary, we demonstrate how the multidimensional spectrum of phenotypic features in *STXBP1*-related disorders can be assessed using a computational phenotype framework to facilitate the development of future precision-medicine approaches.

## Teaser

Xian *et al*. report findings from the largest clinical analysis of *STXBP1*-related disorders to date. Mapping the clinical data of 534 individuals to a computational phenotype framework, the study describes the complex genotype–phenotype landscape of *STXBP1*-related disorders, extending prognostic and mechanistic horizons.

## Introduction

Disease-causing alterations in *STXBP1* are among the most common causes of neurodevelopmental disorders and epilepsy with an estimated frequency of at least 1:30 000.^1,2^ The role of *STXBP1* variants in human neurological disorders was initially identified in Ohtahara syndrome,^3^ but subsequent studies have identified a broader range of other neurological features.^2,4,5^ However, in contrast to other genetic causes of neurodevelopmental disorders,^6,7^*STXBP1*-related disorders have received relatively little attention to understanding genotype–phenotype correlations.

Knowledge about the function of *STXBP1* precedes its discovery as a disease gene. *STXBP1* encodes the syntaxin-binding protein 1, Sec1/Munc18-1, a synaptic vesicle protein involved in vesicle release.^8^ Munc18-1 is a key organizer of the neuronal SNARE complex, the molecular machine driving synaptic vesicle fusion.^9,10^ Homozygous Munc18 knockout mice have no synaptic transmission, emphasizing the crucial role of Sec1/Munc18 in the presynapse.^11^ In addition, the role of *STXBP1* has been recognized in the Golgi complex and intracellular trafficking.^12,13^ The main disease mechanism in *STXBP1*-related disorders is haploinsufficiency.^14^ However, possible dominant-negative effects^15^ and gain-of-function effects^16^ have also been suggested.

Since the initial discovery of *STXBP1*, genetic variants in other genes encoding presynaptic proteins have been found to cause neurodevelopmental disorders, including genes that encode components of the presynaptic fusion machinery such as *SNAP25*, *VAMP2* or *STX1B.*^8,17-19^ Synaptic vesicle recycling also represents a prominent disease mechanism, and we have previously identified disease-causing variants in *DNM1* and *AP2M1* in neurodevelopmental disorders.^20,21^

There are several reasons why research on *STXBP1*-related disorders faces obstacles. First, the overall phenotypic spectrum of *STXBP1*-related disorders is broad, including a wide range of epilepsy presentations and neurological features.^3,22–27^ Second, no clear genotype–phenotype correlations have been identified to date.^2^ Third, as *STXBP1* encodes for a synaptic protein, the pathogenic mechanism linking impaired release of synaptic vesicles to neurological features is less clear than, for example, ion channel-related disorders. Only recently have specific strategies targeting the function of Munc18-1 been suggested. Some of these strategies, such as pharmacological chaperones for protein stabilization, are expected to be assessed in clinical trials soon.^15^

Analysis of clinical features linked to neurodevelopmental disorders has previously been performed in a non-standardized way, using a variety of templates and case-report forms. Methods based on controlled vocabularies and bio-ontologies allow for heterogeneous clinical data to be harmonized across a wide range of sources.^28^ The Human Phenotype Ontology (HPO) is emerging as the *lingua franca* in the field and is widely used in clinical laboratory and research settings.^29,30^ Large studies such as the Deciphering Developmental Disorders study, the UK Biobank, and prominent molecular diagnostic laboratories use HPO, and we have previously shown that heterogeneous phenotypic data in epilepsy disorders can be harmonized and collectively analysed using this framework.^21,31^ Harmonization through HPO terminology also allows for the application of a range of computational approaches, including systematic assessment of overlapping phenotypic features. We have previously demonstrated that methods based on the HPO framework can be used to generate evidence for disease causation by genetic variants in neurodevelopmental disorders, delineate gene-specific phenotypic profiles^31^ and identify phenotypic profiles of recurrent variants within neurodevelopmental genes.^21,32^

Here, we assessed the phenotypic landscape in 534 individuals with *STXBP1*-related disorders, including 281 individuals reported in the scientific literature and 253 individuals recruited through an international network of collaborators. We employed computational approaches utilizing the HPO framework to delineate complex genotype–phenotype relationships in *STXBP1*-related disorders.

## Materials and methods

### Identification of individuals with *STXBP1*-related disorders from literature and existing cohorts

Literature was reviewed to identify all previously reported cases of *STXBP1*-related disorders. We searched PubMed for studies using the search term ‘*STXBP1*’ and identified variants using the Human Gene Mutation Database Professional 2020.2. A total of 253 additional individuals were included through an international collaborative network on *STXBP1*-related disorders, including Children’s Hospital of Philadelphia, USA (*n* = 63, Institutional Review Board #12226); University of Genoa, Italy (*n* = 46); Beijing University First Hospital, China (*n* = 37); University Hospital Heidelberg, Germany (*n* = 21); Hospital Sant Joan de Déu, Spain (*n* = 16); University Medical Center Amsterdam, Netherlands (*n* = 14); Boston Children’s Hospital (*n* = 13); University Hospital Antwerp, Belgium (*n* = 9); Danish Epilepsy Center, Denmark (*n* = 9); Children’s Hospital Colorado, USA (*n* = 8); University of Cambridge, UK (*n* = 6); Sichuan University, China (*n* = 4); Instituto Fernandes Figueira, Brazil (*n* = 4); Newcastle Upon Tyne Hospitals NHS Foundation Trust, UK (*n* = 1); and Samsung Medical Center, Republic of Korea (*n* = 1). We reannotated all missense variants and small indels using a modified annotation pipeline as previously described^21,33^ and only included variants that were either pathogenic or likely pathogenic by American College of Medical Genetics and Genomics criteria (mainly protein-truncating variants, recurrent missense variants) or *de novo* variants in the coding region of *STXBP1*. Regarding copy number variations, we did not include individuals with deletions/duplications involving other genes in addition to *STXBP1*. We assessed 20 individuals that were reported more than once in the literature and 23 individuals contributed by collaborating groups that were previously published in the literature, using the most recent or most complete report to assess phenotypes. Individuals with recurrent variants and insufficient clinical data to distinguish duplicate reports were removed (*n* = 8).

### Mapping of phenotypic features to Human Phenotype Ontology terms

HPO terms were manually assigned by the core research team and collaborators using HPO version 1.2 (release format-version: 1.2; data-version: releases/2018–12-21; downloaded on 02/05/2019). We performed automatic reasoning (propagation) to infer higher-level clinical features based on the ontological structure of the HPO tree as previously reported.^21,31^ We also extracted age of seizure onset and age of last assessment for individuals reported in the literature, when available. We grouped individuals into broad phenotypic categories including Ohtahara syndrome, West syndrome, atypical Rett syndrome, early-onset epileptic encephalopathy (EOEE), other developmental and epileptic encephalopathies (other DEE) and neurodevelopmental disorders based on the initial assignment in the clinical report or the assessment by the contributing clinical group, following a standardized definition applied to the entire cohort (Supplementary material). To compare the frequency of clinical features in specific patient subgroups versus the remainder of the cohort, we used ‘phenograms’ as previously described,^31,32^ highlighting phenotypic features with a nominal *P*-value ≤ 0.05.

### Phenotypic similarity analysis

We used a standard phenotypic similarity assessment^31,34^ to determine whether individuals of a certain subgroup were more related than expected by chance. In brief, phenotypic similarity was assessed by comparing shared clinical features between individuals, weighing the contribution of each feature by its frequency in the overall cohort (Supplementary material). Exact *P*-values were determined by comparing observed versus expected phenotypic similarity based on permutational testing of 100 000 equally sized groups with randomly selected individuals from the larger cohort.

### Reconstruction of epilepsy history including seizure types and seizure frequencies in monthly intervals

To reconstruct seizure history in the Epilepsy Genetics Research Project (EGRP) cohort (Children’s Hospital of Philadelphia, USA, *n* = 62), we retrospectively reviewed clinical records, identifying the presence or absence of seizures within monthly time intervals and coding seizure types using HPO terms. For each monthly interval, we also assessed seizure severity, applying the standard nomenclature for seizure frequency used in the Epilepsy Learning Health System and Pediatric Epilepsy Learning Health System,^35,36^ jointly referred to here as ELHS/PELHS standards. The combined format allowed for the assessment of seizures in three dimensions, including time, seizure type and seizure frequency, and allowed us to determine whether an individual had seizures within each one-month time increment, what type of seizures the individual had and how frequent the seizures were. We then compared seizure frequency as ordinal variables referred to as seizure frequency (SF) scores: multiple daily seizures (>5 per day, SF score = 5), several daily seizures (2–5 per day, SF score = 4), daily seizures (SF score = 3), weekly seizures (SF score = 2), monthly seizures (SF score = 1), no seizures (SF score = 0). When assessing seizure frequency on a group level, we used median seizure frequency and displayed the range of values. For individuals who were reported to have seizures but the seizure frequency was not available from chart review, we assigned the median seizure frequency of other individuals who had seizures in the respective time interval during which the seizure frequency was unknown. While this subcohort only accounted for 12% of the overall cohort, we aimed to leverage the maximum amount of information that could be derived where in-depth longitudinal information was available in the medical records.

### Reconstruction of anti-seizure medications using a hybrid approach of EMR data extraction and manual review

We extracted prescriptions for the most common anti-seizure medications (ASM) from the electronic medical records (EMRs) at Children’s Hospital of Philadelphia.^34^ In several individuals with adrenocorticotropic hormone (ACTH) treatment for infantile spasms, the treatment effect was so rapid that binning into monthly seizure severity scores failed to capture the sequence of medication initiation and resolution of spasms. In these cases, we adjusted the time intervals accordingly to reflect the correct sequence of ACTH administration and seizure improvement. We defined discrete episodes of ASM use as the time between first and last prescription and assumed that ASM use during this episode was uninterrupted. We reviewed all patient charts for the presence of additional ASM and manually added information on prednisolone use for seizure treatment, performed according to the United Kingdom Infantile Spasms Study (UKISS) at our institution,^37^ and the ketogenic diet.^38^ Excluding rescue medications, we compared nine ASM (vigabatrin, topiramate, phenobarbital, levetiracetam, clobazam, UKISS/prednisolone/methylprednisolone, cannabidiol, ACTH and oxcarbazepine) in addition to the ketogenic diet.

### Assessment of comparative anti-seizure medication effectiveness

We assessed the comparative effectiveness of the ASM by analysing the frequency of intervals with seizure improvement coinciding with medication use compared to the frequency of intervals with seizure improvement without the use of the medication. For example, we assessed the number of monthly intervals in which levetiracetam was prescribed and the change in seizure frequency during these intervals based on preceding intervals compared to the change in seizure frequency across intervals in which individuals received a medication other than levetiracetam. We defined reduction in seizure frequency as an improvement or decrease in seizure frequency scores within a monthly interval. For example, an individual with multiple daily seizures (>5 per day, SF score = 5) during 2 months of age that evolved to weekly seizures (SF score = 2) by 3 months of age was defined to have a reduction in seizures. We defined seizure freedom as having no seizures (SF score = 0) across consecutive time intervals. Using this information, we derived odds ratios using Fisher’s exact test for three assessments of comparative effectiveness: (i) the odds ratio of seizure frequency reduction; (ii) the odds ratio of seizure frequency reduction or continuing seizure freedom; and (iii) the odds ratio of continuing seizure freedom only. The latter measure was based on the rationale that some ASM may be initially effective in reducing seizure frequency but not able to maintain continuing seizure freedom.

### Statistical analysis

All computations were performed using the R Statistical Framework.^39^ Statistical testing for the similarity analysis and ASM comparative effectiveness assessment is reported with correction for multiple comparisons using a false discovery rate (FDR) of 5%. Statistical significance was not reached after correction for multiple comparisons in findings reported from longitudinal assessment of clinical features, epilepsy history reconstruction, and analysis on genotype–phenotype correlations between variants. These findings remain on a descriptive level and are presented as odds ratios (OR) with 95% confidence intervals (CI).

### Data availability

Primary data for this analysis are available in the Supplementary material. Computer code for HPO analysis, phenotypic similarity analysis, reconstruction of seizures and ASM history is available at github.com/helbig-lab/STXBP1.

## Results

### The variant spectrum in *STXBP1*-related disorders shows a distinct distribution with several recurrent variants

**Figure 1:**
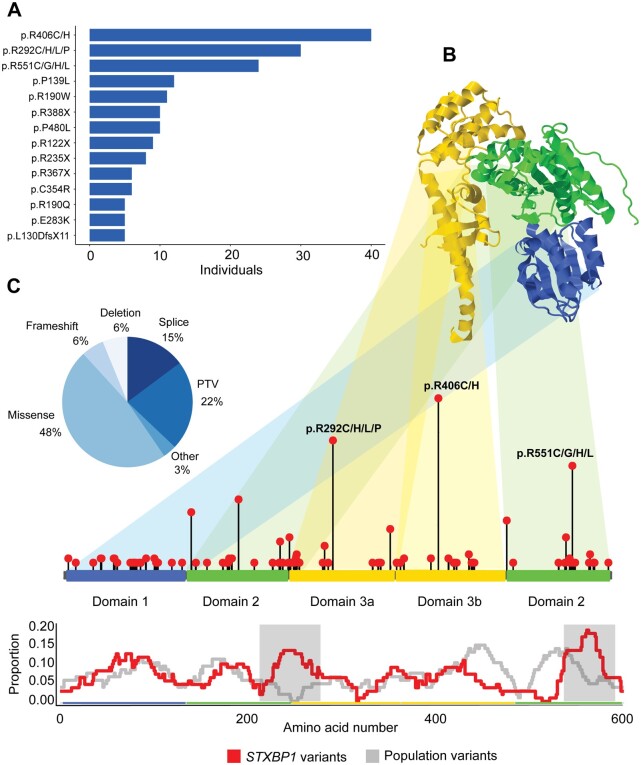
**Overview of *STXBP1* variants.** (**A**) Recurrent *STXBP1* variants found in five or more individuals. (**B**) The STXBP1 protein (*top*) and gene (*bottom*), highlighting a selection of recurrent variant hotspots. The proportion of *STXBP1* disorder-associated versus population variants is shown below, highlighting (grey) regions in which the difference is significant. (**C**) Distribution of variant types. Splice site, frameshift variants and whole and partial gene deletions were included in the PTV/del group.

We analysed the disease-causing variants identified in the 534 individuals included in our study (Fig. 1 and Supplementary Table 1), including 255 individuals with missense variants (108 unique, 25 recurrent variants), 119 individuals with protein-truncating variants (PTV, 79 unique, 12 recurrent variants), 79 individuals with splice site variants (58 unique, 14 recurrent variants), 33 individuals with whole or partial gene deletions, 30 individuals with frameshift variants (24 unique, two recurrent variants), five individuals with duplications, nine individuals with in-frame deletions (six unique, two recurrent variants) and one individual with a synonymous *de novo* variant (Supplementary Table 2). For further analysis, we included splice sites, frameshifts and whole and partial gene deletions in a single group referred to as PTV/del (*n* = 261). Of the 83 novel missense variants found in only one individual, 81 variants were *de novo*, with two variants of unknown inheritance (p.Gly544Cys, p.Glu369Gln) in individuals with epileptic encephalopathy. As these variants occurred at recurrent hotspots, these individuals were included.

We observed 54 recurrent variants (*n* = 271 individuals) in *STXBP1*, including 16 variants found in five or more individuals (Supplementary Table 3). The most common recurrent variants were all arginine residues, p.Arg406His (*n* = 20), p.Arg406Cys (*n* = 20) and p.Arg292His (*n* = 18). Three genomic hotspots were particularly frequent in individuals with *STXBP1*-related disorders, including p.Arg406Cys/His (*n* = 40), p.Arg292Cys/His/Leu/Pro (*n* = 30), p.Arg551Cys/Gly/His/Leu (*n* = 24). In total, recurrent variants identified in five or more individuals accounted for 32% of all individuals with *STXBP1*-related disorders, and the p.Arg406Cys/His alone accounted for 7% of all individuals. When plotted against the frequency of population variants, disease-causing variants were accumulated in two regions (Fig. 1B). These regions do not overlap with known molecular functions.

### The curation of phenotypes in 534 individuals with *STXBP1*-related disorders shows a wide range of clinical features

We included individuals reported in the primary literature (*n* = 281, 53%) and those contributed by participating groups (*n* = 253, 47%). Individuals contributed by the EGRP at Children’s Hospital of Philadelphia were analysed both jointly with the overall cohort and separately with clinical data over time (*n* = 62, see below).

In the joint cohort of 534 individuals, we analysed a total of 5564 initially assigned (base) HPO terms, including 592 unique terms. The most common assigned initial HPO terms were ‘Global developmental delay’ (HP:0001263; *f* = 0.54), ‘Absent speech’ (HP:0001344; *f* = 0.33) and ‘Infantile spasms’ (HP:0012469; *f* = 0.32). The median number of HPO terms assigned to individuals was 10 with a range of 1–53 terms (Table 1). We then performed data harmonization to assign higher-level phenotypic terms, a process referred to as propagation,^32^ which allowed us to reassess the frequency of terms in a manner that gave a better estimate of the true frequency of phenotypic features. After propagation, we obtained a total number of 19 973 HPO terms with 1015 unique terms and a median of 34 terms per individual. The most common HPO terms after propagation were ‘Neurodevelopmental abnormality’ (HP:0012759; *f* = 0.95), ‘Seizures’ (HP:0001250; *f* = 0.89) and ‘Neurodevelopmental delay’ (HP:0012758; *f* = 0.86).

**Table 1:** Cohort information on 534 individuals with *STXBP1*-related disorders

**Demographic information**	
Male	254/534 (47.6%)
Female	236/534 (44.2%)
Not applicable^a^	44/534 (8.2%)
**Age distribution, median (range) [IQR]**	
Age at assessment (*n* = 472)	5 years (0.8 months to 58 years) [2.09 to 12 years]
Seizure onset (*n* = 427)	1.33 months (0.0 to 36 years) [0.24 to 5 months]
Seizure offset (*n* = 80)	9 months (1 month to 42 years) [6 months to 1.6 years]
**Phenotypic information, median (range)**	
Number of phenotypic terms per individual	10 terms (1–53 terms)
Number of phenotypic terms per individual after propagation of HPO terms	34 terms (3–169 terms)
Number of distinct phenotypic terms in cohort	592 terms
Number of distinct phenotypic terms in cohort after propagation of HPO terms	1015 terms

a Literature reports with limited data.

**Figure 2:**
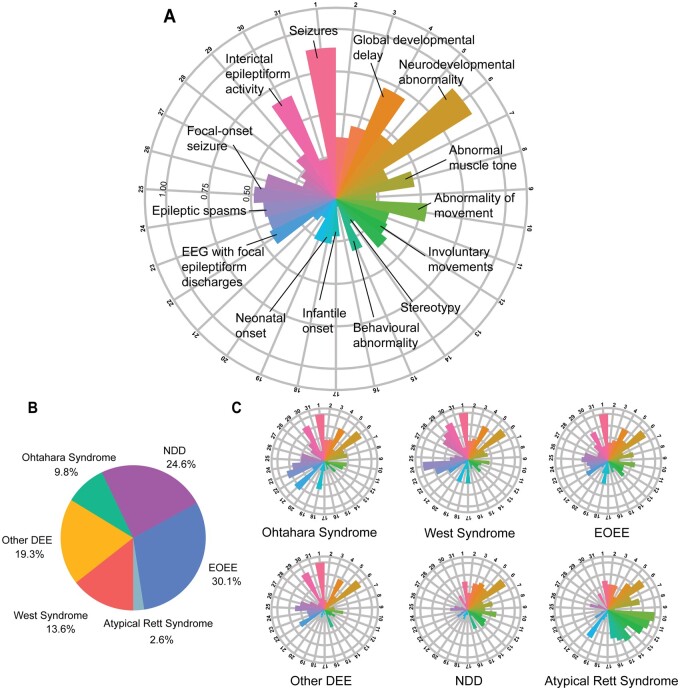
**Individuals reported with *STXBP1*-related disorders and associated phenotypic features across subgroups.** (**A**) Distribution of phenotypic features in the overall cohort of 534 individuals. Radial lines indicate frequency of terms in the respective cohort. 1: seizures; 2: abnormality of higher mental function; 3: delayed speech and language development; 4: global developmental delay; 5: Intellectual disability; 6: Neurodevelopmental abnormality; 7: Neurological speech impairment; 8: Abnormal muscle tone; 9: Abnormality of coordination; 10: Abnormality of movement; 11: gait disturbance; 12: involuntary movements; 13: muscular hypotonia; 14: stereotypy; 15: behavioural abnormality; 16: bruxism; 17: infantile onset; 18: neonatal onset; 19: paediatric onset; 20: sleep disturbance; 21: EEG with burst suppression; 22: EEG with focal epileptiform discharges; 23: EEG with generalized epileptiform discharges; 24: epileptic spasms; 25: focal-onset seizure; 26: generalized-onset seizure; 27: generalized tonic–clonic seizures; 28: hypsarrhythmia; 29: infantile spasms; 30: interictal epileptiform activity; and 31: multifocal epileptiform discharges. (**B**) Individuals were grouped into broad phenotypic groups: EOEE (*n* = 163), neurodevelopmental disorders (NDD, *n* = 129), developmental and epileptic encephalopathy not otherwise specified (other DEE, *n* = 103), West syndrome (*n* = 77), Ohtahara syndrome (*n* = 49) and atypical Rett syndrome (*n* = 13). (**C**) Distribution of phenotypic features across subgroups.

We grouped individuals into six phenotypic categories based on the phenotypes reported in the primary literature or assigned by clinical collaborators (Fig. 2B and Supplementary material). A total of 139 individuals were reported to have specific electroclinical syndromes, including West syndrome (*n* = 77, 15%), Ohtahara syndrome (*n* = 49, 9%) and atypical Rett syndrome (*n* = 13, 2%). The remaining 395 individuals had phenotypes that did not fit the description of West syndrome, Ohtahara syndrome or atypical Rett syndrome and included EOEE (*n* = 163, 31%), neurodevelopmental disorders (*n* = 129, 24%) and developmental and epileptic encephalopathy not otherwise specified (other DEE, *n* = 103, 19%).

Using the harmonized and propagated phenotypic data set, we next assessed the distribution of phenotypes across all six phenotypic groups in the overall cohort (Fig. 2C and Supplementary Table 4). Seizures and neurodevelopmental abnormalities were common across all six groups. The largest difference between phenotypic groups was epileptic spasms that were present in 97% of individuals with West syndrome, 67% of individuals with Ohtahara syndrome, 44% of individuals with EOEE, 33% of individuals with other DEE, 9% of individuals with neurodevelopmental disorders and absent in the atypical Rett syndrome group.

### Most individuals with *STXBP1*-related epilepsy have seizure onset in the first year of life

**Figure 3:**
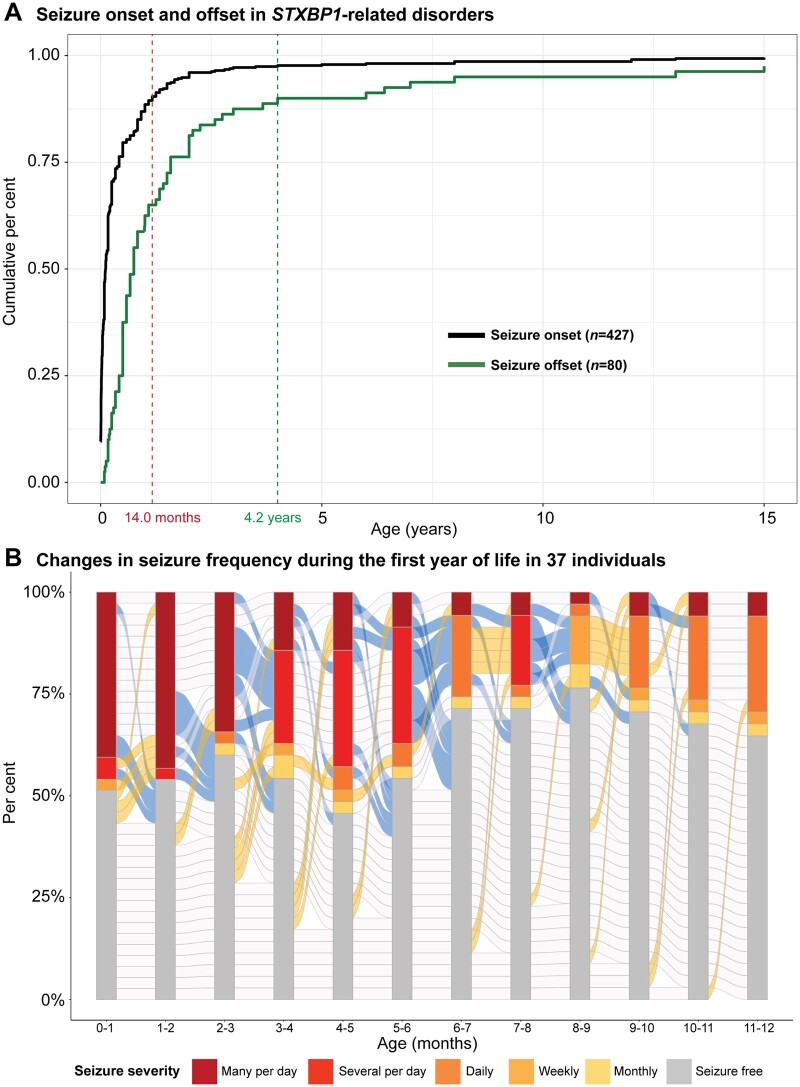
**Seizure patterns in a subset of individuals with *STXBP1*-related disorders.** (**A**) Cumulative distribution of seizure onset in 427 individuals and seizure offset in 80 individuals, highlighting 89% of individuals with seizure onset in the first year of life and offset within the first 5 years. Seizure onset interquartile range (IQR): 0.24–5.0 months. Seizure offset IQR: 5.75 months–1.6 years. (**B**) Changes in seizure frequency in monthly intervals during the first year of life, indicating a dynamic course of seizures in individuals with *STXBP1*. Progression in blue between monthly increments indicates seizure improvement, whereas yellow indicates seizures worsening and grey indicates no change in seizure frequency.

We analysed the epilepsy history of 427/534 individuals with *STXBP1*-related disorders where information on seizure onset and/or offset was available (Fig. 3A). Of all individuals with seizures, 378/427 (89%) had seizure onset during the first year of life. A total of 36 different seizure types were reported in individuals with *STXBP1*-related disorders. ‘Focal-onset seizure’ (HP:0007359; *f* = 0.47), ‘Generalized-onset seizures’ (HP:0002197; *f* = 0.43) and ‘Epileptic spasms’ (HP:0011097, *f* = 0.42) were the most reported seizure types. ‘Simple febrile seizures’ (HP:0011171; *f* = 0.002), ‘Myoclonic atonic seizures’ (HP:0011170; *f* = 0.002), ‘Typical absence seizures’ (HP:0011147; *f* = 0.004) and ‘Hemiclonic seizures’ (HP:0006813; *f* = 0.01) were reported in fewer than 10 individuals with *STXBP1*-related disorders.

Comparing available information on seizure onset, seizure offset and age at evaluation in a subgroup of 62 individuals from the EGRP cohort, we assessed the proportion of individuals with seizures at each time point and the transition between seizure frequencies within each month in the first year of life (Fig. 3B and Supplementary Tables 5 and 6). Seizures were most frequent at the age of 4–5 months (*n* = 19, 54%). While 37 individuals (60%) with *STXBP1*-related epilepsy had seizures at some point within the first year of life, the individual seizure trajectories were heterogeneous. This observation suggests that while seizures in infancy in *STXBP1*-related disorders are common, the patterns can be heterogeneous and often limited to individual patients.

### A subset of clinical features reported in *STXBP1*-related disorders show prominent age dependence

In addition to phenotypic information, we also retrieved the last age of assessment and used frequencies within different age groups to identify clinical features that either increased or decreased over time across four clinically meaningful groups (0–3 years, 3–5 years, 5–11 years, >11 years; Supplementary Table 7). Age-dependence of ‘Intellectual disability’ (HP:0001249) was expected and served as a positive control for this assessment. We observed that the frequency of ‘Tremor’ (HP:0001337), ‘Ataxia’ (HP:0001251), ‘Generalized tonic–clonic seizure’ (HP:0002069), ‘Absence seizure’ (HP:0002121) and ‘EEG with abnormally slow frequencies’ (HP:0011203) increased over time, while the frequency of ‘Hypsarrhythmia’ (HP:0002521) and ‘EEG with burst suppression’ (HP:0010851) decreased over time, as expected. These results suggest a changing clinical picture across the lifespan as phenotypes more frequent in younger individuals such as those with Ohtahara syndrome and West syndrome decreased over time, and phenotypes associated with movement disorders and other types of epilepsy were more frequent in older individuals with *STXBP1*-related disorders.

### PTV/del but not recurrent variants in *STXBP1* show phenotypic similarity

Clinical recognition of phenotypic similarities often depends on the overall constellation and recognizability of phenotypes. We previously demonstrated that clinically intuitive phenotype recognition can be replicated through computational algorithms, referred to as semantic similarity algorithms.^31^ These methods are particularly relevant as associations with single phenotypic features may underestimate clinical similarities within specific patient groups, especially when phenotypic features are rare. In assessing PTV/del versus missense variants, we found that PTV/del (*n* = 261, *P* = 0.015) showed significant phenotypic similarity after correction for multiple testing (FDR 5%). Conversely, when assessing recurrent variants and hotspots, none of the major recurrent variants in *STXBP1* had significant phenotypic similarity, in contrast to main recurrent variants in other neurodevelopmental disorders such as *SCN2A*-related disorders.^32^ This suggests that despite observed trends of recurrent variants with specific phenotypic features (see below), the baseline variability of phenotypic features is too high to allow for discrete clinical subgroups to be recognized. Any variant-specific clinical homogeneity was too subtle to distinguish from the overall heterogeneity in *STXBP1*-related disorders.

### Recurrent variants and PTV/dels in *STXBP1* show phenotypic profiles

**Figure 4:**
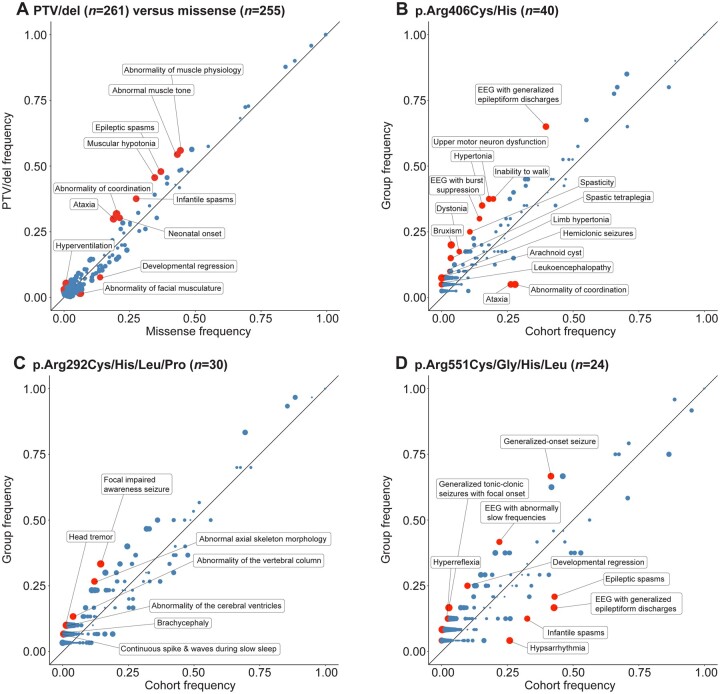
**Phenograms of missense variants versus PTV and most frequent recurrent variant hotspots.** (**A**) Phenogram comparing the frequency of phenotypic features in individuals with PTV/del (*n* = 261) and individuals with missense variants (*n* = 255). PTV/dels included splice sites, frameshifts and whole and partial gene deletions. (**B**) Phenogram comparing the frequency of phenotypic features in individuals with variants in p.Arg406Cys/His and the remainder of the cohort. (**C**) Phenogram comparing the frequency of phenotypic features in individuals with variants in p.Arg292Cys/His/Leu/Pro and the remainder of the cohort. (**D**) Phenogram comparing the frequency of phenotypic features in individuals with variants in p.Arg551Cys/Gly/His/Leu and the remainder of the cohort. Red points indicate HPO terms with uncorrected *P*-values < 0.05, while blue points indicate HPO terms with uncorrected *P*-values ≥ 0.05. Size of points indicate −log_10_(*P*-value).

Based on initial functional data, it has been hypothesized that several recurrent variants may result in either gain-of-function or dominant-negative effects^15,40^ rather than haploinsufficiency, the generally accepted disease-mechanism in *STXBP1*-related disorders. However, when we grouped all variants with known or suspected dominant-negative effects (*n* = 35) including p.Arg406His, p.Pro335Leu, p.Pro480Leu and p.Gly544Asp/Val, statistical significance was not reached with regards to clinical similarity and discrete phenotypic associations after correction for multiple comparisons (Supplementary Fig. 1). Therefore, we examined whether individuals with recurrent variants had phenotypic features that nominally differed from the larger group of individuals with *STXBP1*-related disorders. We used ‘phenograms’ and ‘phenotrees’ as previously described^31,32^ to visually examine the distribution of phenotypes in individuals with recurrent variants (Fig. 4 and Supplementary Fig. 2). We found that p.Arg551Cys/Gly/His/Leu variants were more frequently found in individuals with other DEE (OR 2.65, 95% CI 0.99–6.69); however, none of the other recurrent variants were associated with any electroclinical syndrome or phenotypic group (Supplementary Fig. 3 and Supplementary Table 8).

We then assessed recurrent variants on the level of individual phenotypic features and found that the three most frequent recurrent variant hotspots showed emergent phenotypic profiles. We found that ‘EEG with burst suppression’ (HP:0010851, OR 2.55, 95% CI 1.13–5.46), ‘Spastic tetraplegia’ (HP:0002510, OR 4.92, 95% CI 1.49–14.21) and ‘Inability to walk’ (HP:0002540, OR 2.45, 95% CI 1.15–5.05) were nominally associated with the p.Arg406Cys/His variants (Table 2). The p.Arg292Cys/His/Leu/Pro variants had a higher frequency of ‘Head tremor’ (HP:0002346, OR 7.82, 95% CI 1.24–36.7), ‘Focal impaired awareness seizure’ (HP:0002384, OR 2.94, 95% CI 1.18–6.91), and ‘Abnormality of the cerebral ventricles’ (HP:0002118, OR 6.83, 95% CI 1.11–30.6), while the p.Arg551Cys/Gly/His/Leu variants were nominally associated with ‘Developmental regression’ (HP:0002376, OR 3.06, 95% CI 0.95–8.52), ‘EEG with abnormally slow frequencies’ (HP:0011203, OR 2.53, 95% CI 0.98–6.32) and ‘Generalized-onset seizure’ (HP:0002197, OR 2.81, 95% CI 1.11–7.72). However, none of the associations remained significant after correction for multiple testing. When excluding the major recurrent variants, no domain of the *STXBP1* protein was associated with specific phenotypic features.

**Table 2:** Phenotypic features nominally associated with variant groups

Phenotypic feature (HPO code)^a^	Odds ratio (95% CI)	Group frequency
**Association with protein truncating variants and deletions (PTV/del, *n* = 261)^b^ in at least 20 individuals^c^**		
Abnormality of coordination (HP:0011443)	1.86 (1.22–2.85)	0.32
Ataxia (HP:0001251)	1.84 (1.20–2.84)	0.30
Abnormality of muscle physiology (HP:0011804)	1.59 (1.11–2.29)	0.56
Abnormal muscle tone (HP:0003808)	1.57 (1.09–2.26)	0.54
Muscular hypotonia (HP:0001252)	1.59 (1.10–2.30)	0.46
Epileptic spasms (HP:0011097)	1.57 (1.09–2.27)	0.48
Infantile spasms (HP:0012469)	1.59 (1.08–2.35)	0.38
Neonatal onset (HP:0003623)	1.61 (1.06–2.46)	0.30
Developmental regression (HP:0002376)	0.52 (0.28–0.96)	0.08
**Association with three most frequent recurrent variant hotspots in at least five individuals**		
**p.Arg406Cys/His (*n* = 40)**		
Bruxism (HP:0003763)	6.57 (2.29–17.4)	0.20
EEG with generalized epileptiform discharges (HP:0011198)	2.82 (1.38–5.99)	0.65
Hypertonia (HP:0001276)	2.95 (1.36–6.18)	0.35
Spastic tetraplegia (HP:0002510)	4.92 (1.49–14.2)	0.15
Upper motor neuron dysfunction (HP:0002493)	2.72 (1.28–5.63)	0.38
Inability to walk (HP:0002540)	2.54 (1.15–5.05)	0.38
Spasticity (HP:0001257)	2.77 (1.14–6.23)	0.25
EEG with burst suppression (HP:0010851)	2.55 (1.13–5.46)	0.30
Dystonia (HP:0001332)	2.95 (1.02–5.46)	0.18
**p.Arg292Cys/His/Leu/Pro (*n* = 30)**		
Focal impaired awareness seizure (HP:0002384)	2.94 (1.18–6.91)	0.33
Abnormal axial skeleton morphology (HP:0009121)	2.63 (0.97–6.49)	0.27
**p.Arg551Cys/Gly/His/Leu (*n* = 24)**		
Generalized-onset seizure (HP:0002197)	2.81 (1.11–7.72)	0.67
Developmental regression (HP:0002376)	3.06 (0.95–8.52)	0.25
Epileptic spasms (HP:0011097)	0.35 (0.11–0.99)	0.21
EEG with abnormally slow frequencies (HP:0011203)	2.53 (0.98–6.32)	0.42

a Positive and negative associations for each class by decreasing nominal significance are shown.

b PTV/dels included splice sites, frameshifts and whole and partial gene deletions.

c All nominal associations with PTV/del are shown in Supplementary Table 10.

Finally, we assessed phenotypic profiles associated with PTV/dels and missense variants (Table 2). We found that individuals with PTV/dels in *STXBP1* were nominally associated with West syndrome (OR 1.60, 95% CI 0.95–2.73) and phenotypic features including ‘Infantile spasms’ (HP:0012469, OR 1.59, 95% CI 1.08–2.35) and ‘Ataxia’ (HP:0001251, OR 1.84, 95% CI 1.20–2.84), while individuals with missense variants were more likely to have other DEE (OR 2.05, 95% CI 1.28–3.32; Supplementary Table 9). We did not observe additional differences in missense variants versus PTV/del between the other phenotypic groups (Supplementary Fig. 4).

### Reconstruction of seizure frequency in monthly increments demonstrates a complex pattern

**Figure 5:**
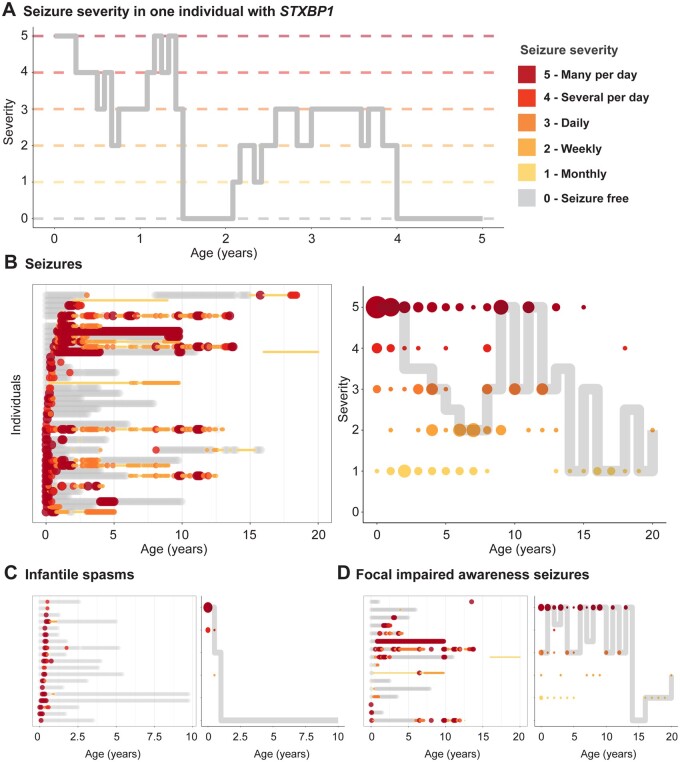
**Reconstruction of seizure frequency in monthly increments.** (**A**) Changes in seizure severity with time in one individual with *STXBP1*-related disorder are shown. Seizure severity is represented by ordinal seizure frequency (SF) scores: multiple daily seizures (SF = 5), several daily seizures (SF = 4), daily seizures (SF = 3), weekly seizures (SF = 2), monthly seizures (SF = 1), no seizures (SF = 0). (**B**) Pattern of seizure frequency in individuals with seizures, regardless of type (HP:0001250, *n* = 43). Seizure frequencies of individuals tracked across time (*left*), each row along the *y*-axis representing a unique individual. Colour and size of dots represent seizure severity. Median severity in the subgroup with size of dots indicating number of individuals (*right*). (**C**) Pattern of seizure frequency in individuals with infantile spasms (HP:0012469, *n* = 19). (**D**) Pattern of seizure frequency in individuals with focal impaired awareness seizures (HP:0002384, *n* = 16).

For the 62 individuals in the EGRP cohort, we were able to retrospectively analyse clinical data and assess the presence or absence of seizures in monthly time increments. We used the HPO framework to assess seizure types, including additional documentation of seizure frequencies. Accordingly, we reconstructed monthly seizure types and seizure frequencies in a standardized and computable format (Fig. 5A). In total, we were able to reconstruct seizure histories for 4433 total months across 62 individuals.

We first assessed the temporal evolution of seizure frequency in individuals independent of their age of seizure onset (Fig. 5B). We found that seizure frequency was highest in the first year of life with 91% of all individuals with seizures presenting with multiple daily seizures. Seizure frequency across all individuals decreased dramatically by the age of 7 years. Median seizure frequency increased by the age of 10 years but was only based on clinical data from a limited number of individuals and we were unable to draw conclusions regarding the cause of seizures worsening. We observed that the patterns for seizure frequencies were different for specific seizure types, including ‘Infantile spasms’ (HP:0012469) and ‘Focal impaired awareness seizure’ (HP:0002384, Fig. 5C and D).

### Assessment of seizure frequency and anti-seizure medication prescription allows for a limited comparative effectiveness framework

**Figure 6:**
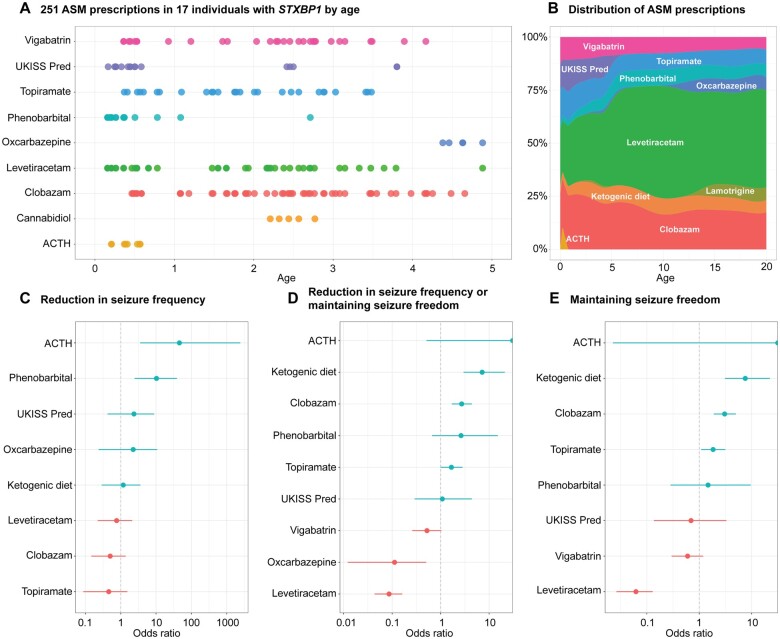
**Comparative effectiveness assessment of ASM and treatment responses.** (**A**) Selection of ASM prescriptions extracted from the EMR, across ages 0–5 years in a subset of individuals with *STXBP1*. (**B**) Extension of **A**, showing the distribution of individuals on ASM and ketogenic diet across ages 0–20 years. (**C**) Relative effectiveness of ASM in reducing seizure frequency. (**D**) Relative effectiveness of ASM in reducing seizure frequency or maintaining seizure freedom. (**E**) Relative effectiveness of ASM in maintaining seizure freedom. Odds ratios were calculated based on changes in seizure frequency to ASM use across monthly time intervals.

For a subset of the EGRP cohort (*n* = 17), medical care was provided in a single centre where EMR could be mined to extract prescriptions and administrations of ASM (Fig. 6A and B). After correction for multiple testing, we found that phenobarbital (*n* = 7, OR 10.3, 95% CI 2.47–38.9) and ACTH (*n* = 4, OR 45.9, 95% CI 3.55–2434) were more likely to reduce seizure frequency than to worsen or to have no effect (Fig. 6C). When assessing the ability of specific ASM to reduce seizure frequency or maintain seizure freedom, we found that clobazam (*n* = 8, OR 2.72, 95% 1.70–4.41) and the ketogenic diet (*n* = 3, OR 7.19, 95% 2.98–21.1) were effective (Fig. 6D and E).

We next examined patterns in ASM response to specific ages and seizure types. We found that all significant ASM responses were primarily restricted to the first 2 years of life (Supplementary Fig. 5) and specific to unique seizure types. The ACTH response could be attributed to improvements in infantile spasms and the phenobarbital response was due to reduced seizure frequency of focal seizures (Supplementary Fig. 6). These combinations of medication response and seizure types reflected the common use of these ASM in clinical practice, indicating that responses in *STXBP1*-related disorders may be seizure type-specific rather than dependent on the underlying genetic aetiology.

## Discussion

We analysed the phenotypic landscape of *STXBP1*-related disorders using a harmonized data set of 19 973 individual phenotypic annotations in 534 individuals. We highlighted common phenotypic patterns in individuals with *STXBP1*-related disorders, emphasized the genetic architecture characterized by recurrent variants with specific phenotypic associations and outlined the dynamic seizure patterns in *STXBP1*-related disorders.

### Data harmonization in a heterogeneous neurodevelopmental disease

*STXBP1*-related disorders pose a challenge in describing the clinical phenotype and outlining the phenotypic spectrum. Since the initial description by Saitsu *et al*.,^3^ various phenotypes have been reported throughout the literature. However, the existing literature has only included a limited number of individuals and provided a heterogeneous amount of clinical data in case-cohort descriptions. We attempted to overcome this challenge by capturing clinical data through a data harmonization framework using HPO terminology. The HPO is emerging as a common data standard in the medical genetics field and enables the description of clinical features at various levels of detail. Using the HPO framework, we combined this heterogeneous information into a joint analysis that included all individuals reported in the literature and previously unreported individuals contributed by 15 collaborating centres, allowing us to assess the distribution of over 1000 unique phenotypic terms in individuals with *STXBP1*-related disorders.

### Age-related features in *STXBP1*-related disorders

Confirming prior studies, nearly all individuals with *STXBP1*-related disorders have neurodevelopmental features, including developmental delay and/or intellectual disability, emphasizing the relevance of developmental trajectories when examining *STXBP1*-related disorders. As expected, we found that intellectual disability is diagnosed in relation to patient age, with a frequency of almost 90% of individuals above the age of 11, including 64% of individuals with sufficient clinical data to classify severe or profound intellectual disability and only 2% with mild intellectual disability. In addition, at least 38% of individuals were non-verbal in the age group above 11 years, highlighting the role of communication in *STXBP1*-related disorders. We acknowledge that our study design did not allow for the dissection of developmental trajectories. In future studies, prospective assessments using standardized outcome assessments of patient-level developmental features will be paramount, including assessment of non-verbal measures of cognition.

Several age-dependent features in *STXBP1*-related disorders may be underappreciated in the existing literature given the wide range of ages of individuals reported in the literature and recruited at collaborating clinical centres. For example, tremor and ataxia were identified in up to 40% of individuals above the age of 11 years, emphasizing that features such as movement disorders may be more common in older individuals than previously expected and may require further scrutiny in future studies focusing on the adult phenotypes of *STXBP1*-related disorders.

### Seizure phenotypes in *STXBP1*-related disorders

We found that 89% of seizures in *STXBP1*-related disorders start in the first year of life. Accordingly, once individuals with *STXBP1*-related disorders reach the second or third year of life, the risk for future seizures decreases with a probability of future seizures of 5% or less by the age of 3 years based on data available from clinical reports. However, we acknowledge that long-term outcomes for most individuals are not available and that the true risk for seizure recurrence in *STXBP1*-related disorders remains unknown.

Infantile spasms were found in almost half of all individuals with *STXBP1*-related disorders who have seizures, a clinical feature that stands out within the context of the wider range of neurodevelopmental disorders. We have previously shown that within a group of 663 individuals with various developmental and epileptic encephalopathies assessed across almost 3500 cumulative patient-years, *STXBP1*-related disorders represent the only genetic aetiology significantly associated with infantile spasms.^34^ The overall high frequency of infantile spasms may open up a window for rapid identification of individuals with *STXBP1*-related disorders for precision medicine trials.

### Genotype–phenotype correlations for *STXBP1* recurrent variants

Historically, *STXBP1*-related disorders are considered to present without clear genotype–phenotype correlations.^2^ For example, in contrast to ion channel disorders, where both gain-of-function and loss-of-function variants frequently result in vastly different phenotypes with distinct clinical features, medication responses and specific protein domains, *STXBP1*-related disorders were not observed to present with clear identifiable genomic features and clinical subgroups.

In clinical practice, genotype–phenotype associations in *STXBP1*-related disorders are subtle and evade the identification of distinct clinical constellations in individuals with shared molecular features. Our study demonstrates the benefits of harmonizing phenotypic data in a large collaborative cohort to identify differences in the risk of developing certain phenotypes in individuals with particular hotspot variants. Given that the emerging phenotypic groups may reinforce different underlying disease mechanisms, further work to outline clinical subgroups in *STXBP1*-related disorders will be paramount. Surprisingly, none of the major recurrent variants in *STXBP1* showed significant phenotypic similarity, in contrast to the main recurrent variants in other neurodevelopmental disorders such as *SCN2A*-related disorders.^32^ This lack of phenotypic similarity for recurrent variants indicates that when compared to the phenotypes of *STXBP1-*disorders as a whole, the baseline variability of phenotypic features is too high for single-phenotype associations to result in overall phenotypic similarity. This negative result mirrors clinical experience and suggests that phenotypic patterns in *STXBP1*-related disorders are often not distinct enough to be recognized as subgroups.

However, emerging functional data suggest dominant-negative mechanisms for some of the recurrent *STXBP1* variants, including p.Arg406His, p.Pro335Leu, p.Pro480Leu and p.Gly544Asp/Val.^15^ Accordingly, we assessed whether specific phenotypic features were nominally correlated with hotspot variants in *STXBP1*. We found that features associated with EOEE before the typical age of infantile spasms, including EEG with suppression burst, were increased by a factor of two in individuals with p.Arg406Cys/His recurrent variants. Likewise, the frequency of spastic tetraplegia was increased almost 5-fold, while ataxia was less common as in the larger group of individuals with *STXBP1*-related disorders by more than 7-fold. Other recurrent variants were found to have nominal associations with distinct phenotypes, including a 3-fold increase in focal impaired awareness seizures among individuals with p.Arg292/Cys/His/Leu/Pro variants. In contrast, the frequency of infantile spasms was reduced more than 3-fold among individuals with p.Arg551Cys/Gly/His/Leu variants. None of the clinical features found to be associated with recurrent variants were exclusive to either carriers of recurrent variants or the larger population of individuals with *STXBP1*-related disorders. However, grouping all variants with known or suspected dominant-negative mechanisms did not result in statistically significant clinical similarity. This suggests that *STXBP1*-related disorders with presumed or known dominant-negative effects do not have a joint, recognizable phenotypic signature that sets this group of variants apart in our study.

### Genotype–phenotype correlations for missense variants versus PTV/del

We found that individuals with PTV/dels were twice as likely to have infantile spasms, hypsarrhythmia and ataxia, and were more likely to have hypotonia and neonatal seizure onset than individuals with missense variants. While these single genotype–phenotype associations did not remain significant after correction for multiple testing, we found that individuals with PTV/dels were significantly more phenotypically similar than expected by chance. We believe that this information will help delineate strategies to stratify patients for future clinical studies by identifying individuals more likely to have specific variant classes as well as provide valuable information to our patients’ families.

### Heterogeneous patterns of seizures in *STXBP1*-related disorders

Despite the high frequency of infantile spasms, seizures in individuals with *STXBP1*-related disorders in the first year of life are not uniform. While seizures are most common between the ages of 4 and 5 months, reconstructing seizure types and seizure frequencies revealed a dynamic and heterogeneous pattern of seizure onset and offset in the first year of life. For example, during each month, only 40% of all individuals with seizures at some point during infancy had seizures, and the remaining 60% either had seizures that had occurred earlier, started later, or were temporarily controlled. This observation emphasizes the need for longitudinal assessments in future studies and clinical trials. *STXBP1*-related disorders represent a paradigmatic neurodevelopmental condition with heterogeneous phenotypic features that occur and remit over time, and these methods will allow for comparison with other neurodevelopmental disorders.

### Implications for clinical management of individuals with *STXBP1*-related disorders

In our study, we aimed to leverage the entire existing clinical information on *STXBP1*-related disorders. We highlighted numerous new disease-causing variants within *STXBP1* that have not previously been reported in the literature. Our report of all known variants will allow for formal reclassification of these variants for individuals with uncertain diagnoses and highlight hotspots of recurrent variants that require further scrutiny with respect to the disease mechanism. Furthermore, this genetic information coupled with a phenotypic map of clinical phenotypes will allow clinicians to obtain a better understanding of the full range of clinical features to be expected in *STXBP1*-related disorders, including age-dependent features and phenotypes that are conspicuously absent. For example, status epilepticus, a common feature in many other DEEs, has a very low frequency in *STXBP1*-related disorders in our study. By assessing the true frequency of clinical features within subgroups (e.g. variant types) and across all individuals with *STXBP1*-related disorders, clinicians can better counsel patients on the relative risk of developing certain clinical features, such as the increased risk of infantile spasms in individuals with PTV/del in *STXBP1*, as well as reference an expected time frame for developmental milestones and allow for early intervention.

The reconstruction of seizures histories in a subset of individuals provides an in-depth overview of the changing picture of *STXBP1*-related seizures, particularly during the first year of life when individuals are often misdiagnosed, and testing and treatment is frequently delayed. By demonstrating that *STXBP1*-related disorders have a highly dynamic seizure pattern with temporary seizure resolution, we provide clinicians with information that provides anticipatory guidance in the management of *STXBP1*-related epilepsy in the first year of life when seizure onset is observed in >90% of individuals. The detailed reconstruction of seizure presentations will allow for more specific estimates and counselling of seizure risks over time as a child ages, including seizure onset and offset.

Finally, we provide information from a subset of individuals on treatment response in *STXBP1*-related disorders. Even though the number of individuals where information was available was limited, we found significant differences with respect to the effects of various anti-seizure medications that were dependent on seizure type and age. This information may provide clinicians with initial evidence with respect to effective therapies, including the beneficial effect of the ketogenic diet in maintaining seizure freedom. We hope that our comparative effectiveness analysis will serve as a guiding framework for learning health systems and future approaches in understanding drug effects in *STXBP1*-related disorders and the genetic epilepsies.

### Limitations

Our study draws upon a large and heterogeneous amount of clinical information in a complex neurodevelopmental disorder. As we included all clinical information available in the literature and through many collaborating centres, the degree of phenotypic completeness and available information is variable. For example, different centres can have different diagnostic practices that can lead to potential detection bias. In addition, clinical features are documented at varying levels of specificity. However, this also presented us with the opportunity to utilize the HPO to leverage the various levels of detail and assess the overall landscape of clinical features in *STXBP1*-related disorders, overcoming the attentional bias found in traditional case-cohort reports. In summary, we present a descriptive study, outlining the complex clinical presentations in *STXBP1*-related disorders based on all available information within the literature and across an international network of collaborating centres.

Accordingly, our work includes three levels of data: a larger cohort with phenotypic information and limited longitudinal data (*n* = 534), a smaller cohort of individuals from a single centre where longitudinal data were available (*n* = 62) and a subset of this cohort where ASM could be extracted from the medical records (*n* = 17). We acknowledge that the smaller number of individuals in these subgroups can limit the conclusions that can be drawn. Nevertheless, the information drawn from these subcohorts provides valuable insights with regards to temporal evolution of seizures (*n* = 62, 370 patient years) and response to ASM (*n* = 17, 48 patient years), providing a baseline for future studies on natural history and therapy response in *STXBP1*-related disorders, in which the developmental trajectory is relatively unknown and targeted pharmacological therapies are still being developed.

## Conclusion

*STXBP1*-related disorders represent a perplexing group of neurodevelopmental disorders in which the broad range of neurodevelopmental features poses a conceptual difficulty in outlining phenotypic subgroups, genotype–phenotypic correlations and assessing drug response. In addition, the rapidly changing seizure frequencies, particularly in the first year of life, add additional complexity when trying to define shared clinical trajectories. We addressed these complexities through a computational approach coupled with data harmonization through biomedical ontologies. We show that these methods allowed us to outline specific phenotypic patterns in *STXBP1*-related disorders associated with protein-truncating variants and deletions and recurrent missense variants. We hope that the results from our study will guide the design and outcome measures of future precision medicine trials in *STXBP1*-related disorders, one of the most common developmental and epileptic encephalopathies.

## Supplementary Material

awab327_Supplementary_Data

## Abbreviations

ACTH: adrenocorticotropic hormone
ASM: anti-seizure medications
DEE: developmental and epileptic encephalopathy
EOEE: early onset epileptic encephalopathy
HPO: Human Phenotype Ontology
PTV: protein-truncating variants
